# Role of oral bacteria composition and functional gene profiles in respiratory diseases

**DOI:** 10.1136/bmjresp-2025-003938

**Published:** 2026-06-18

**Authors:** Christine Cramer, Ian Philip George Marshall, Michael J. Abramson, Nils Oskar Jõgi, Maryia Khomich, Shyamal D. Peddada, Bente Sved Skottvoll, Vivi Schlünssen, Randi Jacobsen Bertelsen

**Affiliations:** 1 Department of Public Health, Research Unit for Environment, Danish Ramazzini Centre, Aarhus University, Aarhus, Denmark; 2 Department of Occupational Medicine and Department of Otorhinolaryngology, Head & Neck Surgery, Aarhus University Hospital, Aarhus, Denmark; 3 Department of Biology, Section for Microbiology, Aarhus University, Aarhus, Denmark; 4 School of Public Health and Preventive Medicine, Monash University, Melbourne, Victoria, Australia; 5 Department of Medical Sciences, Uppsala University, Uppsala, Sweden; 6 Department of Clinical Science, University of Bergen, Bergen, Norway; 7 Biostatistics and Computational Biology Branch, National Institute of Environmental Health Sciences, Durham, North Carolina, USA; 8 Oral Health Centre of Expertise in Western Norway, Bergen, Norway

**Keywords:** Microbiota, Asthma, Asthma Epidemiology, Respiratory Function Test, Allergic lung disease

## Abstract

**Introduction:**

The oral microbiome has been shown to be associated with respiratory health, primarily in adult case studies or among children. This relationship has been scarcely investigated in adult population-based cohorts.

**Objectives:**

To investigate the association between oral microbiome and respiratory health, more specifically asthma, chronic rhinosinusitis (CRS), lung function and fractional exhaled nitric oxide (FeNO) in a population-based cross-continental multicentre study among adults.

**Methods:**

Subgingival samples from 355 adult European Community Respiratory Health Survey participants from Norway, Australia and Estonia underwent metagenomic sequencing. Respiratory disease was defined from questionnaires and sensitisation from specific immunoglobulin E (IgE)/skin prick tests. Spirometry and FeNO were measured. The associations between alpha diversity and disease status were evaluated in cross-sectional analyses using logistic regression adjusting for sex, smoking and study centre. Differential abundance analyses were performed using analysis of compositions of microbiomes with bias correction.

**Results:**

Alpha diversity differed by study centre and sensitisation status and was associated with non-allergic CRS (richness: 1.12, 95% CI 1.03 to 1.22). A similar though not statistically significant pattern was seen for forced vital capacity (FVC) below the lower limit of normal (LLN). *Lachnospiraceae* and *Xanthomonas* were more abundant in the oral microbiome of non-asthmatics and individuals without CRS, respectively, as compared with asthmatics and CRS patients. Several functional genes (1477–3391) and genera (54-98) were only present in the non-case groups, whereas individuals with affected respiratory health had 0–74 unique functional genes, but no unique genera present only in their respective groups.

**Conclusion:**

Increased alpha diversity was associated with non-allergic CRS and a similar trend was seen for FVC below LLN. Bacterial composition and functional profiles of the oral microbiome differed by respiratory health status. This study is novel in exploring functional gene profiling in relation to asthma and FeNO.

**WHAT IS ALREADY KNOWN ON THIS TOPIC**

- In adults, the oral microbiome has been sparsely investigated in relation to respiratory health, especially in population-based cohorts. Existing research has rarely included international cohorts or functional profiling.

**WHAT THIS STUDY ADDS**

- It highlights the importance of geographically diverse study populations and emphasises the importance of investigating composition and functional profiles. It suggests functional redundancy could be seen in 'healthy' microbiome.

**HOW THIS STUDY MIGHT AFFECT RESEARCH, PRACTICE OR POLICY**

- Functional redundancy in the microbiome could be target of future preventative measures, should they prove important. Chronic rhinosinusitis, when investigated, should take sensitisation into account.

## Introduction

 Asthma is a heterogeneous disease of the lower airways characterised by inflammation, variable respiratory symptoms and airflow limitation.^1^ The prevalence of asthma is increasing worldwide, with an estimated 400 million patients by 2025.^2^ Chronic rhinosinusitis (CRS) is linked to asthma under the united airways theory but is less studied.^3^

Spirometry is used to evaluate individuals with respiratory symptoms suspected of having asthma or chronic obstructive pulmonary disease (COPD), to monitor the effects of treatment and to evaluate overall respiratory health.^4^ Also, fractional exhaled nitric oxide (FeNO), a measure of eosinophilic airway inflammation, is linked to asthma, lung function decline and immunoglobulin E (IgE)-mediated sensitisation.^5 6^

The oral microbiome contains more than 700 microbial species and is the second largest microbial community in humans after the gut and is influenced by geography and environment.^7 8^ The oral microbiome is intertwined with the lung microbiome through the oral-lung axis and may act as a biomarker for phenotyping of asthma.^9 10^

The oral microbiome has been suggested to impact respiratory health, but the evidence is inconclusive.^8^ Most studies looking into associations between the oral microbiome and respiratory health have been case studies investigating children with lung disease. In adults, the impact of oral microbiome on respiratory health has been sparsely investigated, especially within population-based cohorts. However, previous studies do suggest a link between the oral microbiome, lung function, asthma, CRS and sensitisation.^11^,^13^

Another important aspect of the oral microbiome is its functional profile, which has been suggested to differ between individuals with and without CRS based on predictive modelling.^12^ However, to our knowledge, no studies have explored sequenced functional genes in relation to adult asthma, CRS or FeNO.

We aimed to investigate how the oral microbiome was associated with respiratory health in three geographically separated populations in Europe and Australia.

## Methods

### Study population

In total, 355 participants were recruited from the third wave (2010–2013) of the multicentre population-based European Community Respiratory Health Survey (ECRHSIII)^14^ limited to three centres with gingival sampling: Bergen (Norway): n=121, Melbourne (Australia): n=125 and Tartu (Estonia): n=107. The study population is reflective of the ECRHS cohort in being a random sample, with a symptomatic subsample.

Data were retrieved from interviewer-administered questionnaires and lung function, FeNO measurements and gingival samples from the clinical examination. The ECRHSIII main questionnaire and the clinical protocol are available on the ECRHS website (www.ecrhs.org). Patients and the public were not involved in the design or conduct of this study.

### Oral microbiome

Gingival fluid was collected concurrently at each study centre by jointly trained personnel following standard operating procedures using sterile materials. The gingival fluid sampling was performed with sterile mirror and tweezers, sterile gloves and surgical face mask. The samples were subsequently transported on dry ice to a −80 °C freezer for centralised extraction and sequencing. Extraction and metagenomic sequencing were performed by Clinical Microbiomics (Copenhagen, Denmark) collectively for all samples across study centres to eliminate the risk of a batch effect. We profiled the taxonomic composition with MetaPhlAn V.4.1.0 (CHOCOPhlAnSGB marker gene database vJun23).^15^ The annotation of microbial genes to specific biological pathways was enabled through the Kyoto Encyclopedia of Genes and Genomes (KEGG) database. Each gene was mapped to the EggNOG (V.5.0).^16^ See details in online supplemental methods.

Alpha diversity was assessed using richness (number of species-level genome bins) and the Shannon index. Beta diversity was evaluated using the abundance-based Bray-Curtis dissimilarity.

### Outcomes

Disease status was derived from the questionnaires collected during the clinical examination concurrently with gingival sampling. Both asthma and CRS status were based on questions regarding the last 12 months indicating current disease.

*Asthma* was defined by having had an asthma attack in the last 12 months and/or current use of asthma medication in line with previous ECRHS studies.^17^

We defined *CRS* as an affirmative answer to at least two of these questions (all for symptoms lasting more than 12 weeks during the last 12 months), one of them having to be either (1) or (3): (1) blocked nose; (2) pain or pressure around the forehead, nose or eyes; (3) discoloured nasal discharge or discoloured mucus in the throat; (4) reduced or absent sense of smell. This is in line with the European Position Paper on Rhinosinusitis and Nasal Polyps criteria and previous studies.^18^

Individuals were classified as *sensitised* if they had a specific IgE ≥0.35 kU_A_/L (allergens: *Dermatophagoides pteronyssinus*, Timothy grass, cat) or a positive skin prick test (allergens: Timothy grass, ragweed, *D. pteronyssinus*, cat, birch, *Blatella*, olive, *Alternaria*, dog, *Cladosporium herbarum*, *Parietaria, D. farinae*). If no data on sensitisation were available from ECRHSIII, information on IgE from ECRHS I or II was used.

*Spirometry* was performed by trained staff using an EasyOne spirometer (ndd Medical Technologies, Andover, Massachusetts, USA) in accordance with the recommendations of the American Thoracic Society (ATS) and the European Respiratory Society (ERS).^19^ We collected data on forced expiratory volume in the first second (FEV_1_) and forced vital capacity (FVC). Global Lung Initiative (GLI) 2012 reference equations^20^ were used to calculate z-scores. The GLI reference equations account for age, height, gender and ethnicity. Z-score ≤−1.64 was used to identify subjects below the lower limit of normal (LLN).

*FeNO* was measured using NIOX MINO (Aerocrine AB, Solna, Sweden) according to guidelines at the plateau of expiration.^21^ FeNO was dichotomised into <25 ppb or ≥25 ppb in line with clinical practice guidelines.^22^

### Covariates

A priori covariates considered were age, sex, study centre and smoking (current, ex or never smokers). Smoking and sex are well-known risk factors for asthma and CRS and both conditions also vary with age and country.^1 23^ In comparative analyses performed by Clinical Microbiomics using the data of this study, the diversity of the oral microbiome was shown to vary significantly with smoking status and age (results not shown). All covariates were recorded at the time of clinical examination.

Apart from being a separate outcome, sensitisation status was used as a proxy for allergic disease status.

### Statistics

Alpha diversity was analysed using multiple logistic regression. Beta diversity was analysed using the analysis of similarity test on the Bray-Curtis distance from the vegan package V.2.6.8, where in pairwise unadjusted analyses, individuals were compared by outcome status.^24 25^

Analysis of compositions of microbiomes with bias correction (ANCOM-BC2) from the package ANCOMBC V.2.4.0 was used to test for bacterial genera or functional genes having structural zero groups compared pairwise by outcome status. After removing the taxa or functional genes with structural zeroes or a prevalence of <10%, ANCOM-BC2 determined which KEGG functions or bacterial taxa were differentially abundant between the outcome groups while controlling for other covariates.^26 27^ All p values were adjusted for multiple testing with the Holm-Bonferroni method and all reported results passed the ANCOM-BC2 sensitivity (SS) filter. Significance was assessed with a threshold of false discovery rate <0.05. ANCOM-BC2 was also used to determine which bacterial taxa were differentially abundant between centres in unadjusted analyses.

In the adjusted analyses, we included sex, age, smoking and study centre. Analyses were performed as complete case analyses and therefore the number of individuals varied across tables.

Statistical analyses were performed in R V.4.3.2 (2023–10-31 ucrt).^28^

## Results

DNA sequencing library preparation was successful for 335 (94%) samples: Bergen (n=119), Melbourne (n=107) and Tartu (n=109). The average richness of the oral microbiome was 140 and Shannon Index 3.5.

### Study participants

The proportion of individuals in the cohort sampled randomly versus based on respiratory symptoms varied (random sample: 15% (Bergen), 83% (Tartu) and 70% (Melbourne)) (table 1). The median age for the overall population was 53 years [range 40–65 years] and half were men. The sex and age distribution differed between centres, as did the prevalence of sensitisation (55% (Melbourne) and 20% (Tartu)) and respiratory diseases (eg, asthma 37% (Bergen) and 12% (Tartu)). Across centres, the median FeNO was 16 ppb. More than 84% had lung function above the LLN.

**Table 1:** Characteristics of the participants by centre

	Bergen, n=119	Tartu, n=109	Melbourne, n=107	Total, n=335
	N (%)	N (%)	N (%)	N (%)
Sex				
Men	70 (59)	37 (34)	59 (55)	166 (50)
Age, year				
Mean (SD)	53 (7.1)	51 (6.9)	56 (6.0)	53 (6.9)
Sample				
Random	18 (15)	90 (83)	75 (70)	183 (55)
Symptomatic	100 (84)	19 (17)	32 (30)	151 (45)
Smoking status				
Smoker	36 (30)	19 (17)	7 (7)	62 (19)
Ex-smoker	42 (35)	20 (18)	38 (36)	100 (30)
Never	40 (34)	70 (64)	62 (58)	172 (51)
Sensitisation status				
Sensitised	57 (48)	22 (20)	59 (55)	138 (41)
Asthma				
Yes	44 (37)	12 (11)	14 (13)	70 (21)
Chronic rhinosinusitis				
Yes	24 (20)	6 (6)	3 (3)	33 (10)
FVC, l				
Mean (SD)	4.4 (1.1)	4.0 (0.96)	4.2 (1.1)	4.2 (1.1)
FEV_1_, l				
Mean (SD)	3.2 (1.0)	3.1 (0.75)	3.1 (0.82)	3.2 (0.87)
FeNO (ppb)				
Median (IQR)	13 (12)	13 (8)	23 (16)	16 (13)
Richness (SGBs)				
Mean (SD)	120 (60)	170 (110)	110 (57)	140 (64)
Shannon Index				
Mean (SD)	3.4 (0.68)	3.9 (0.52)	3.2 (0.70)	3.5 (0.70)

For some variables, there were missing data; more for the objective measures (up to 7.3% for sensitisation status in Tartu) than for the questionnaire data (<1%).

FeNO, fractional exhaled nitric oxide; FEV_1_, forced expiratory volume in the first second; FVC, forced vital capacity; SGB, species-level genome bin.

**Figure 1:**
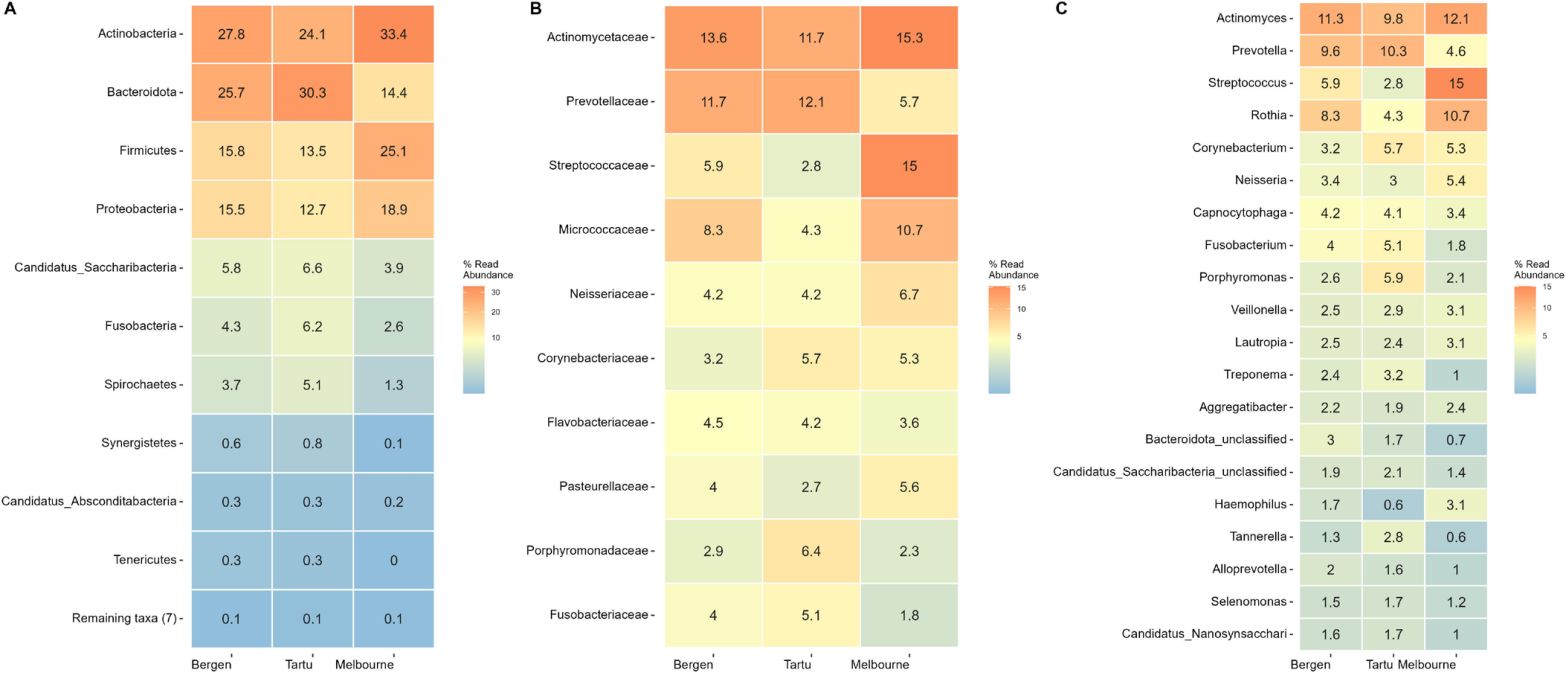
Heatmap grouped by centre, colour-coded by % of the specific taxa present within each study centre. (**A**) 10 most prevalent phyla, (**B**) 10 most prevalent families and (**C**) 20 most prevalent genera.

The most frequently found phylum in the gingival samples was *Actinobacteria* in Bergen and Melbourne and *Bacteroidota* in Tartu (figure 1). The *Prevotella* genus accounted for 9.3% of reads in Bergen and 10.3% in Tartu, but only 4.6% in Melbourne. *Streptococcus* was more frequently found in samples from Melbourne (15%), as compared with Tartu (2.8%) and Bergen (5.9%). The genus *Rothia* was more prevalent in samples from Melbourne (10.7%) and Bergen (8.3%), than Tartu (4.3%). *Porphyromonas* accounted for 2.6% of reads in Bergen, 2.1% in Melbourne and 5.9% in Tartu.

### Diversity

**Figure 2:**
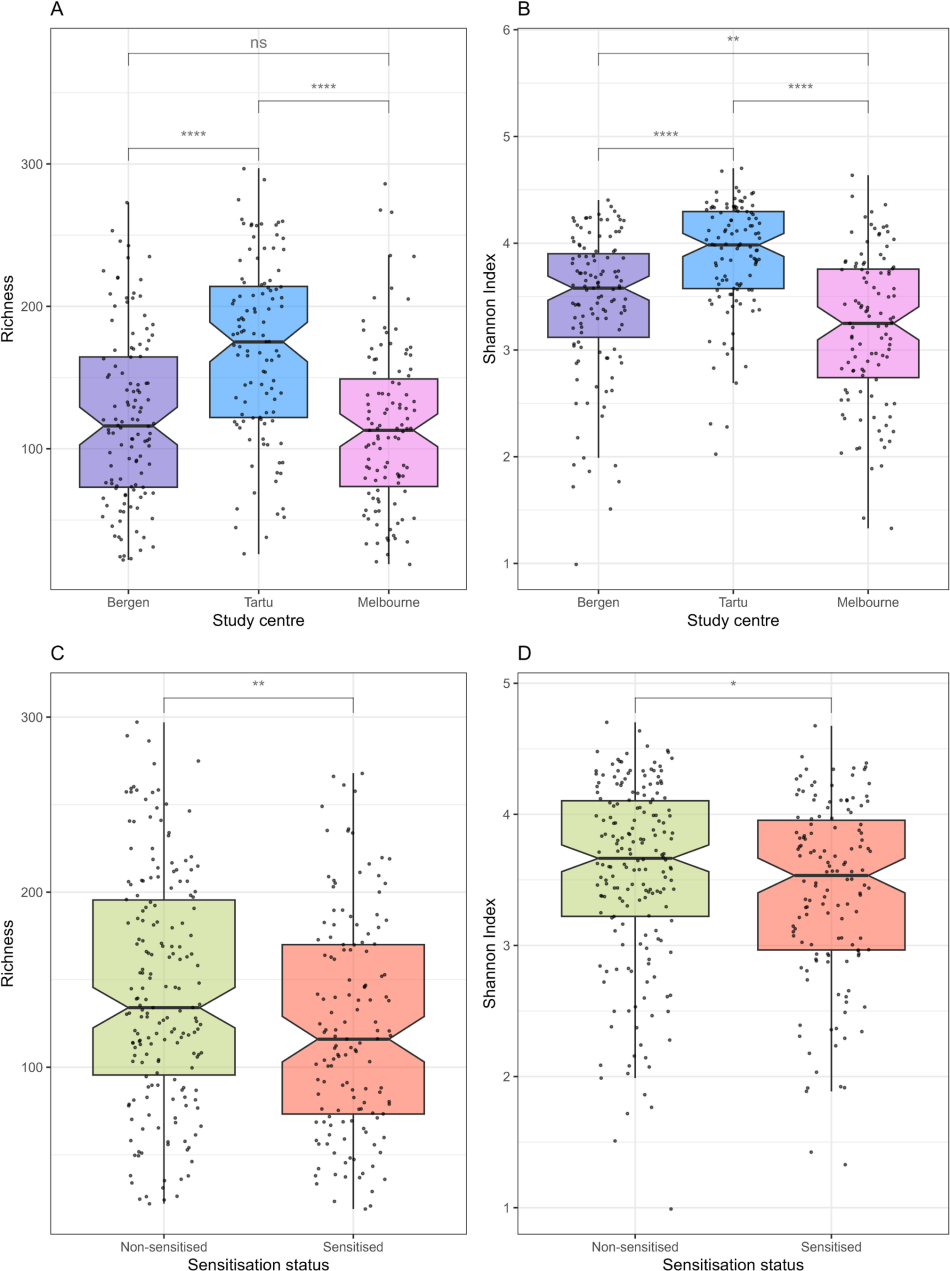
Box plots showing alpha diversity grouped by centre and sensitisation. Richness (**A**) and Shannon Index (**B**) in relation to study centre. Richness (**C**) and Shannon Index (**D**) in relation to sensitisation status. Differences in medians are estimated by Wilcoxon rank-sum test (p values: ns ≥0.05, * <0.05, ** 0.01, *** <0.001, **** <0.0001).

Alpha diversity was higher in Tartu, compared with Melbourne and Bergen (figure 2A,B), and diversity was higher among non-sensitised compared with sensitised individuals (figure 2C,D). In stratified analyses (online supplemental figure S1), the tendency of lower diversity of the oral microbiome in the sensitised group remained for Tartu and Bergen, but no difference was seen for Melbourne. The stratified results were expectedly all non-significant due to power issues.

In the adjusted models, the OR for CRS increased with increasing alpha diversity (richness: 1.08, 95% CI 1.01 to 1.16; Shannon Index: 1.87, 0.98–3.88) (table 2). A similar pattern was seen for FVC Z-score below LLN with increased OR slightly attenuated in the adjusted analysis (richness: 1.08, 95% CI 1.00 to 1.17; Shannon Index: 1.97, 0.87–5.25). Sensitisation tended to be negatively associated with richness (unadjusted: 0.95, 95% CI 0.92 to 0.98; adjusted: 0.98, 0.94–1.02). Results for FVC Z-scores and sensitisation did not reach statistical significance in adjusted analyses.

**Table 2:** Logistic regression analyses of microbial alpha diversity as related to sensitisation, asthma, CRS, lung function and FeNO

	Sensitisation		Asthma		CRS		FVC Z-score below LLN		FEV_1_ Z-score below LLN		FeNO ≥25 ppb	
Alpha diversity	OR (95% CI)	P value	OR (95% CI)	P value	OR(95% CI)	P value	OR (95% CI)	P value	OR (95% CI)	P value	OR (95% CI)	P value
Richness (/10 SGBs)												
Unadjusted	0.95 (0.92 to 0.98)	0.01	0.98 (0.94 to 1.02)	0.32	1.04 (0.99 to 1.10)	0.14	1.09 (1.02 to 1.17)	0.01	1.05 (1.00 to 1.11)	0.04	0.98 (0.94 to 1.02)	0.39
Adjusted*	0.98 (0.94 to 1.02)	0.40	1.01 (0.96 to 1.06)	0.78	1.08 (1.01 to 1.16)	0.02	1.08 (1.00 to 1.17)	0.06	1.02 (0.97 to 1.07)	0.53	1.02 (0.97 to 1.07)	0.53
Shannon Index												
Unadjusted	0.76 (0.55 to 1.04)	0.08	1.11 (0.76 to 1.65)	0.60	1.49 (0.86 to 2.74)	0.18	2.65 (1.23 to 6.55)	0.02	1.58 (0.96 to 2.75)	0.09	0.77 (0.54 to 1.11)	0.16
Adjusted*	1.04 (0.73 to 1.50)	0.83	1.39 (0.89 to 2.22)	0.16	1.87 (0.98 to 3.88)	0.07	1.97 (0.87 to 5.25)	0.17	1.06 (0.61 to 1.91)	0.85	1.06 (0.68 to 1.68)	0.80

* Adjusted for age, sex, study centre and smoking (never, ex or current). Lung function analyses were not adjusted for sex and age, since this was taken into account as part of the Z-scores.

CRS, chronic rhinosinusitis; FeNO, fractional exhaled nitric oxide; FEV_1_, forced expiratory volume in the first second; FVC, forced vital capacity; SGB, species-level genome bin.

Increased richness and Shannon Index were associated with non-allergic CRS (richness: 1.12, 95% CI 1.03 to 1.22; Shannon Index: 2.04, 0.89–5.36), but not allergic CRS, in adjusted analyses (online supplemental table S1).

No difference in beta diversity was seen between outcome groups, using pairwise comparisons (online supplemental tables S2 and S3). There was a significant difference in beta diversity between the centres (R2=6.7%, p=0.001) (see online supplemental figure S2).

### Differentially abundant taxa and functional genes

Eight genera were differentially abundant between Tartu and Bergen with a q-value cut-off of 0.05 in unadjusted analyses. Between Bergen and Melbourne, it was 15 genera, and between Tartu and Melbourne, 37 genera were differentially abundant (see online supplemental table S4).

Out of the 291 identified genera in the whole population, 79 were unique to non-asthmatics as compared with asthmatics, 98 were unique to individuals without CRS as compared with individuals with CRS and 54 were unique to individuals with FeNO <25 ppb as compared with individuals with FeNO ≥25 ppb. In comparison, *no* genera were exclusively found in samples from individuals with any of the outcomes investigated (table 3).

**Table 3:** Number of bacterial genera or functional genes unique to either cases or non-cases

	Number of individuals in the analysis (cases)	Genera *unique* to individuals with the outcome (n)	Genera *unique* to individuals without the outcome (n)
Outcome			
Asthma	334 (70)	0	79
CRS	335 (33)	0	98
FeNO ≥25 ppb	326 (76)	0	54

		Functional genes *unique* to individuals with the outcome (n)	Functional genes *unique* to individuals without the outcome (n)
Outcome			
Asthma	334 (70)	16	2633
CRS	335 (33)	0	3391
FeNO ≥25 ppb	326 (76)	74	1477

The outputs of the ANCOM-BC2 analyses are available on request from the corresponding author.

ANCOM-BC2, analysis of compositions of microbiomes with bias correction; CRS, chronic rhinosinusitis; FeNO, fractional exhaled nitric oxide.

Out of the 10 991 identified functional genes in the whole population, 2633 were unique to non-asthmatics, 3391 only in individuals without CRS and 1477 only in individuals with FeNO <25 ppb as compared with their respective non-cases. 16 functional genes were unique to asthmatics and 74 only in individuals with FeNO ≥25 ppb, but none were unique for CRS (table 3).

Most of the genera and functional genes unique to the oral microbiome of individuals with or without outcome were present in <10% of the samples within the group (online supplemental figure S3). Using a cut-off q-value of 0.05, three bacterial genera were more abundant in non-asthmatics compared with asthmatics (unclassified *Lachnospiraceae* (log fold change (LFC): −1.11), GGB1833 (LFC: −1.17) and unclassified *Candidatus Absconditabacteria* (LFC: −1.22)), and one bacterial genus was more abundant in individuals without CRS compared with those with CRS (*Xanthomonas* (LFC: −1.39)) (online supplemental figure S4 and online supplemental table S5). No genera were more abundant in asthmatics or CRS cases, compared with their respective non-cases, and no genera were differentially abundant with FeNO ≥25 ppb.

Several functional genes were differentially abundant by outcome status with a q-value cut-off of 0.05 (online supplemental figure S5): 27 (non-asthmatics), three (non-CRS) and one (FeNO<25 ppb). The genes represented a wide variety of functions. No functional categories were more common in individuals with asthma, CRS or FeNO ≥25 ppb.

## Discussion

This study examined different dimensions of respiratory health within a diverse population to provide a more comprehensive understanding of the association between respiratory health and the oral microbiome. Microbial diversity was associated with CRS and FVC, to some extent with sensitisation, but not with asthma or FeNO. Several genera and biological functions of the oral microbiome were differentially abundant between individuals with or without impaired respiratory health.

The geographical difference in composition and diversity of the oral microbiome supports previous findings by Lin *et al* of geographical variation of sputum microbiome in COPD patients across China.^7^ However, it has previously been reported that the geographical variability of the sputum microbiome was limited.^29^ This highlights the importance of including geographically diverse individuals in future studies on oral microbiome. However, caution should be taken as to the generalisability of the results, without being verified in a separate validation cohort.

A limitation of this study is the lack of information regarding recent antibiotic use, which could influence the oral microbiome. However, recent systemic antibiotic use was not expected to be very prevalent in this community cohort. This is corroborated by a study from another ‘westernized’ country (USA) concurrently with the data collection in this study, suggesting a prevalence of 3%.^30^ Socioeconomic status was not included in the adjusted analyses, but smoking status is expected to at least in part act as a crude proxy for this. Overall, we do not expect residual confounding to substantially influence the association between the oral microbiome and respiratory health outcomes in the current study.

Differences in the microbiome between study centres could be caused by differences in sampling technique or varying degrees of contamination to which low-biomass samples are vulnerable.^8^ However, a rigorous standard operating procedure, jointly trained sampling personnel, sterile procedures and materials for sampling, as well as centralised extraction and sequencing, made this explanation unlikely.

When the ECRHS clinical cohort was established in 1990, it was enriched with symptomatic participants. The population for this study is based on this cohort and is thus enriched with individuals with respiratory symptoms, especially for the Bergen cohort, since gingival sampling started late, after most of the random sample had been examined. For this reason, the frequencies of respiratory disease, sensitisation, abnormal lung function and FeNO were not representative of the general population. However, we do not expect the sampling strategy 20 years prior to the current study to influence the association between oral microbiome and respiratory health/lung function. Questionnaire-based outcomes are at risk of misclassification, but any misclassification would likely be non-differential and bias towards the null. Since both asthma and CRS were based on questions regarding the prior 12 months, recall bias should be limited. Finally, we performed cross-sectional analyses baring us from making conclusions on causality, but the analyses are, however, well-suited to investigate short-term effects between the oral microbiome and current symptoms and clinical outcomes like FeNO and lung function.

In part due to the sampling, the three centres exhibited significant differences in the proportions of smokers and individuals with sensitisation, factors that could influence the composition and diversity of the oral microbiome.^31^ Interestingly, despite these variations, the centres did not cluster in a manner consistent with smoking prevalence (highest in Bergen) or sensitisation rates (significantly lower in Tartu) when evaluating composition. Rather, Bergen and Tartu grouped together, suggesting that geographical proximity or comparable living conditions trumped these characteristics in determining the composition of the oral microbiome.

When examining alpha diversity, Bergen clustered with Melbourne, displaying lower diversity than Tartu. This pattern aligned with the descriptive analyses and to some extent the logistic regression analysis, indicating reduced diversity among sensitised individuals. When stratifying the descriptive results for centre, the tendency of lower diversity of the oral microbiome in the sensitised group remained for the two European centres; however, no difference was seen for Melbourne. We could speculate that a reason for the difference between the European centres and Melbourne could have been a difference in the local relevance of the allergen panel used to define sensitisation in this study. However, this is not expected to have greatly affected our sensitisation estimates, as Melbourne was the centre with the highest sensitisation prevalence. In contrast to our descriptive analyses, no difference in alpha diversity was found between sensitised individuals and either non-sensitised asthmatics or non-sensitised healthy controls in a study by Durack *et al*.^11^ Durack *et al* investigated 66 individuals and used 16S rRNA sequencing of oral wash and induced sputum to sample the oral microbiome. Thus, the difference in results between the two studies could be explained by methodological differences.

The most frequent phyla in Bergen and Tartu were *Actinobacteria* and *Bacteroidota*, and for Melbourne, *Firmicutes* and *Proteobacteria. Actinobacteria*, *Bacteroidota* and *Firmicutes* have previously been found to contribute to 88% of reads in sputum samples of healthy individuals.^32^ An asthma-associated genus such as *Streptococcus* was more differentially abundant in the oral microbiome of the Australian participants compared with participants from the two Northern European centres in unadjusted analyses. *Rothia*, commonly found in the oral cavity, known to have an airway anti-inflammatory effect, was more differentially abundant in the oral microbiome of the Melbourne participants compared with the participants from Tartu.^11 33^

Most studies on oral microbiome of adults in relation to respiratory health are case-control or case-only studies, and only two population-based studies exist to our knowledge, both based on a Norwegian cohort with 16S rRNA analyses of the microbiome.^13 34^

We found no consistent association between alpha diversity of the oral microbiome and sensitisation or asthma. Perez-Garcia *et al* found decreased alpha diversity to be associated with asthma exacerbations in their case-only study, suggesting that incorporating asthma severity into future studies could be informative.^33^ Increased alpha diversity was found to be associated with low FVC, in line with a study on patients with idiopathic pulmonary fibrosis.^35^ However, Shigdel *et al* found no association between richness or Shannon index and FVC below LLN in a general Norwegian study population.^13^

No association was found between FEV_1_ or FeNO and any of the alpha diversity indices. In a small case-control study, Durack *et al* found higher diversity of oral wash to be associated with increased FEV_1_, but no association between induced sputum microbiome and FEV_1_, underlining the importance of sample site when comparing results.^11^

CRS did show an association with alpha diversity indices. The only prior study on oral microbiome and CRS in adults, a case-control study by Yuan *et al* with 18 cases, reported no association between alpha diversity of salivary microbiome and CRS.^12^ To our knowledge, no prior studies have distinguished between allergic and non-allergic CRS in relation to microbiome. A possible explanation for why we found an association with CRS but not with asthma in the current study could be that CRS prevalence increased more with age, possibly making CRS more temporally relevant for the current oral microbiome as compared with asthma, which has a broader age at onset (childhood, adult and late onset).^18 36^ While both asthma and CRS were based on questions regarding the past 12 months, CRS was based on symptoms and asthma was based on medication use and asthma attacks. This could also cause the CRS definition to indicate more ‘active’ disease. Regarding the distinction between allergic and non-allergic CRS, a similar pattern of diverging directions has been seen in adult farmers regarding microbial components and allergic and non-allergic asthma.^37^

We found that *Lachnospiraceae* was more abundant in the oral microbiome of non-asthmatics compared with asthmatics, when adjusting for sex, age, smoking and study centre. According to preclinical in vivo models, *Lachnospiraceae* can alleviate inflammatory and allergic diseases by modulating the immune system.^38^ In contrast, Marri *et al* found the phylum *Bacillota* to be more common in induced sputum samples of asthmatics compared with non-asthmatics.^39^

*Xanthomonas* was more abundant in individuals without CRS than individuals with the condition, and this is the first time, to our knowledge, that this genus has been linked to health and disease in humans. *Xanthomonas* is pathogenic to plants and associated with spoilage of fresh produce and has been found in drinking water.^40^ Yuan *et al* found controls’ saliva enriched with *Cardiobacterium* and *Neisseria* as compared with saliva of CRS patients, both of which belong to the same phylum as *Xanthomonas*.^12^

A major strength of this study is the resolution and KEGG annotation of metagenomic sequencing and the analyses of microbiome-related functional pathways in this study present novel findings of a decrease in functions in the oral microbiome of asthma, CRS and elevated FeNO compared with non-asthmatic individuals without CRS and FeNO <25. No prior studies have, to our knowledge, reported similar analyses for asthma or FeNO. Yuan *et al* found an increase in functional pathways of, for example, transporters and porphyrin metabolism in CRS patients using prediction software, but we found no functions to be increased in or unique to the microbiome of individuals with CRS when sequencing the genes.^12^ The significant number of functional genes unique to and the increased abundance of functional genes in the oral microbiome of non-cases could be seen as an example of *functional redundancy*, assisting in upholding stability of the healthy microbiome, which has previously been described for gut microbiota.^41^

## Conclusion

In this study, increased richness of the oral microbiome was associated with a higher prevalence of non-allergic CRS and to some extent with a higher prevalence of impaired FVC and a lower prevalence of sensitisation. No consistent association was observed between alpha diversity and asthma, regardless of allergic status. Similarly, no consistent relationships were found between alpha diversity and FEV_1_ and FeNO.

Four bacterial genera were significantly differentially abundant across asthma status and CRS presence, and several bacteria were unique to non-cases. Overall, the oral microbiome of individuals with asthma, CRS or elevated FeNO had functional genes that were absent or less abundant compared with those without these conditions.

## Supplementary material

10.1136/bmjresp-2025-003938 online supplemental file 1

## Data Availability

Data are available upon reasonable request.
