# Supplementary material for: Role of oral bacteria composition and functional gene profiles in respiratory diseases
Source: BMJ Open Respir Res. 2026 Jun 18;13(1):e003938. doi: 10.1136/bmjresp-2025-003938 (PMC13289405; doi:10.1136/bmjresp-2025-003938)
Supplement: online supplemental file 1 [file bmjresp-13-1-s001.pdf]

Methods (evaluating the oral microbiome)

Sampling was done at each study centre, but subsequent analyses have been performed by our group or by Clinical Microbiomics (Copenhagen, Denmark). Clinical Microbiomics carried out the extraction, library preparation, DNA sequencing, gene mapping, filtration of host DNA and Kyoto Encyclopedia of Genes and Genomes (KEGG) orthology annotations.

Gingival samples

Gingival fluid was collected during the clinical visit using sterile paper points F4 and F5 (PROTAPER, Jacobsen Dental). The paper points were placed in the gingival crevice at five sites in the lower and upper jaw where they were held in place for five seconds. Five paper points from the upper, and five from the lower jaw, were pooled in two separate sterile 2 mL safelock Biopur tubes. Samples were stored without buffer at -80°C.

DNA extraction

DNA was extracted from pooled gingival fluid samples from the upper or lower jaw (n=5) using the NucleoSpin 96 Soil (Macherey-Nagel) kit with horizontal bead beating on a Vortex-Genie 2 (2700 rpm for 5 minutes). A positive mock community control from ZymoBIOMICS Microbial Community Standard (Zymo Research) and minimum one negative control was included per batch of samples from the DNA extraction and throughout the laboratory process including sequencing.

Shot-gun sequencing

DNA extract quality and quantity was evaluated by agarose gel electrophoresis and Qubit 2.0 fluorometer quantitation, respectively. Ultrasonication was used to randomly shear DNA into fragments of around 350 bp. To assess the prepared libraries and the fragment size distribution Qubit 2.0 fluorometer quantitation and Agilent 2100 Bioanalyzer were used.

Library construction with fragmented DNA was done using NEBNext Ultra II Library Prep Kit for Illumina (New England Biolabs). The prepared DNA libraries were evaluated with Qubit 2.0 fluorometer quantitation and Agilent 2100 Bioanalyzer for the fragment size distribution. Quantitative real-time PCR was used to determine the concentration of the final library before sequencing. The library was sequenced using 2 x 150 bp paired-end sequencing on an Illumina platform.

Pre-processing, taxonomic, and functional profiling of metagenomic samples

Clinical Microbiomics removed host reads by mapping raw FASTQ files to the human reference genome GRCh38 with Bowtie2 (v. 2.4.2)[1].

Taxonomic profiling

We pre-processed the filtered non-host FASTQ files running fastp v.0.23.4 with default settings[2]. Sample sequence quality was assessed before and after pre-processing running FastQC v.0.11.9 followed by multiQC v.1.20 to summarize the FastQC reports[3,4]. We profiled the taxonomic composition with MetaPhlan v.4.1.0 (CHOCOPhlAnSGB marker gene database vJun23) with default parameters and additionally parameter -t rel_ab_w_read_stats to include both read counts and relative abundance estimates[5]. All MetaPhlan4 sample outputs were merged into one relative abundance table. Available R-scripts from MetaPhlAn4 were run to make a phylogenetic tree file in Newick format, calculate alpha-diversity metrics and beta-diversity distances.

Functional profiling

Clinical microbiomics trimmed non-host reads to remove adapters and bases with Phred score <20 using AdapterRemoval (v. 2.3.1)[6]. Read pairs in which one or both were below 100 bp were discarded.

The Clinical Microbiomics Human Oral Ho01 gene catalog (8 554 254 genes), was created from 706 non-public deep-sequenced human saliva samples, 1305 oral samples compiled from 21 publicly available data sets, and 1326 publicly available genome assemblies from microbial strain isolates and used as reference gene catalogue.

Retained reads after pre-processing were mapped to the Human Oral Ho01 gene catalogue using BWA mem (v. 0.7.17)[7], with mapping quality ≥ 20 and read alignment of ≥ 95 % identity over ≥ 100 bp. A read was considered unmapped if > 10 bases of the read did not align to the gene or extend beyond the gene. Reads meeting the alignment length and identity criteria but not the mapping quality threshold were considered multi-mapped. A gene count table was created with the number of uniquely mapped read pairs, where either one or both individual reads were uniquely mapped to a gene, for each gene.

EggNOG-mapper (v. 2.0.1) was used to map each gene in the Human Oral Ho01 gene catalog to the EggNOG (v. 5.0) orthologous groups database, resulting in KEGG orthology (KO) database annotations for 46% of genes[8]. Functional potential profiles based on KOs were calculated as the proportion of all mapped reads that mapped to a given KO.

References

1. Langmead B, Salzberg SL. Fast gapped-read alignment with Bowtie 2. Nature methods. 2012 Apr;9(4):357–9.
2. Chen S. Ultrafast one-pass FASTQ data preprocessing, quality control, and deduplication using fastp. iMeta. 2023 May 1;2(2):e107.
3. Andrew S. FastQC: A Quality Control Tool for High Throughput Sequence Data [Online]. 2010.
4. Ewels P, Magnusson M, Lundin S, Käller M. MultiQC: summarize analysis results for multiple tools and samples in a single report. Bioinformatics. 2016 Oct 1;32(19):3047–8.
5. Blanco-Míguez A, Beghini F, Cumbo F, McIver LJ, Thompson KN, Zolfo M, et al. Extending and improving metagenomic taxonomic profiling with uncharacterized species using MetaPhlAn 4. Nature Biotechnology 2023 41:11. 2023 Feb 23;41(11):1633–44.
6. Schubert M, Lindgreen S, Orlando L. AdapterRemoval v2: rapid adapter trimming, identification, and read merging. BMC research notes. 2016 Feb 12;9(1).
7. Li H, Durbin R. Fast and accurate short read alignment with Burrows-Wheeler transform. Bioinformatics (Oxford, England). 2009 Jul;25(14):1754–60.
8. Huerta-Cepas J, Forslund K, Coelho LP, Szklarczyk D, Jensen LJ, Von Mering C, et al. Fast Genome-Wide Functional Annotation through Orthology Assignment by eggNOG-Mapper. Mol Biol Evol. 2017 Aug 1;34(8):2115–22.

Table S1. Logistic regression analyses of microbial alpha diversity by allergic and non-allergic asthma and CRS

	**Allergic asthma**		**Non-allergic asthma**		**Allergic CRS**		**Non-allergic CRS**	
Alpha diversity indices	*OR# (95% CI)*	*P-value*	*OR# (95% CI)*	*P-value*	*OR# (95% CI)*	*P-value*	*OR# (95% CI)*	*P-value*
Richness (/10 SGBs)								
Unadjusted	0.97 (0.92 – 1.02)	0.30	0.99 (0.93 – 1.06)	0.85	0.97 (0.88 – 1.06)	0.49	1.09 (1.02 – 1.17)	0.02
Adjusted*	1.01 (0.95 – 1.07)	0.72	1.01 (0.94 – 1.08)	0.87	1.00 (0.89 – 1.12)	0.98	1.12 (1.03 – 1.22)	0.01
Shannon Index								
Unadjusted	1.01 (0.64 – 1.66)	0.96	1.21 (0.69 – 2.26)	0.53	1.13 (0.51 – 2.87)	0.78	1.68 (0.82 – 3.87)	0.18
Adjusted*	1.35 (0.79 – 2.41)	0.29	1.29 (0.68 – 2.62)	0.46	1.29 (0.50 – 4.02)	0.63	2.04 (0.89 – 5.36)	0.12

Odds ratio

* Adjusted for age, sex, study centre and smoking (never, ex, or current)

Table S2. Beta diversity of the oral microbiome and respiratory outcomes

	**Sensitisation**		**Asthma**		**Chronic rhinosinusitis**		**FVC Z-score below LLN**		**FEV_1 Z-score below LLN**		**FeNO ≥ 25ppb**	
Beta diversity	*R*	*P-value*	*R*	*P-value*	*R*	*P-value*	*R*	*P-value*	*R*	*P-value*	*R*	*P-value*
ANOSIM												
Unadjusted	-0.004	0.623	*-0.018*	*0.717*	*-0.104*	*0.993*	-0.135	0.994	-0.061	0.939	0.046	0.054

Table S3. Beta diversity of the oral microbiome and respiratory outcomes taking sensitisation into account

	**Allergic asthma**		**Non-allergic asthma**		**Allergic chronic rhinosinusitis**		**Non-allergic chronic rhinosinusitis**	
Beta diversity	*R*	*P-value*	*R*	*P-value*	*R*	*P-value*	*R*	*P-value*
ANOSIM								
Unadjusted	-0.083	0.982	-0.024	0.669	-0.110	0.935	-0.054	0.813

Table S4. Output from ANCOM-BC2 analyses of differentially abundant genera between study centres

Bergen vs Tartu		
**taxon**	**lfc_cityTartu**	**q_cityTartu**
g__Alkalibacterium	-1,779781767	2,6161E-08
g__Candidatus_Nanosynbacter	-1,151525217	0,000249847
g__Enterococcus	-1,025826341	0,003965639
g__Lachnospiraceae_unclassified	1,33101876	1,06574E-05
g__Leptotrichia	1,035768259	0,003264964
g__Paracoccus	-0,950141051	0,002063926
g__Pelagibacterium	-2,635114084	1,67901E-15
g__Xanthomonas	-3,532616207	2,04602E-18
**Bergen vs Melbourne**		
**taxon**	**lfc_cityMelbourne**	**q_cityMelbourne**
g__Alkalibacterium	-1,489714481	0,000132947
g__GGB1025	-1,068038696	0,008183277
g__GGB10852	-1,172977746	0,002225943
g__GGB1611	-0,932802445	0,025466463
g__GGB2663	-1,250048084	0,000514131
g__Hallella	-1,061957684	0,009916515
g__Metamycoplasma	-1,457636326	2,49351E-05
g__Parabacteroides	3,891148786	2,68248E-21
g__Parvimonas	-1,052445759	0,027200439
g__Pelagibacterium	-1,036605132	0,044224965
g__Peptidiphaga	1,013001979	0,035321694
g__Scardovia	-2,777998734	1,67852E-13
g__Streptococcus	1,285342303	0,000891835
g__Treponema	-1,151298583	0,007799628
g__Xanthomonas	-2,274693211	1,7256E-09
**Tartu vs Melbourne**		
**taxon**	**lfc_cityMelbourne**	**q_cityMelbourne**
g__Anaerolineaceae_unclassified	-1,181951594	0,000552472
g__Campylobacter	-0,842149173	0,045130216
g__Candidatus_Nanosynsacchari	-1,163875551	0,005735184
g__Desulfobulbus	-1,115298041	0,0006751
g__Dialister	-1,446915981	3,47579E-06
g__Enterococcus	1,861319774	2,92314E-09
g__Eubacteriales_Family_XIII_Incertae_Sedis_unclassified	-1,031230271	0,004242251
g__Exiguobacterium	1,026153478	0,001468125
g__Filifactor	-0,991728912	0,004193444

g__Fretibacterium	-1,025300354	0,001084991
g__Fusobacterium	-1,220341467	0,000214289
g__GGB1022	-1,088598423	0,002285266
g__GGB10852	-1,562306139	1,97963E-07
g__GGB12798	-1,275836669	0,000116074
g__GGB2663	-1,285679475	5,61612E-05
g__GGB2666	-1,636829525	1,39137E-07
g__GGB4300	-0,914758872	0,024807354
g__GGB4308	-0,94168206	0,019161998
g__GGB49400	-1,148763599	0,000364593
g__GGB49434	-1,470634898	7,32387E-06
g__GGB71076	-1,921382487	1,9565E-08
g__Granulicatella	1,301951877	0,000552472
g__Haemophilus	1,807936833	7,61669E-06
g__Hallella	-1,135765014	0,00089853
g__Lachnospiraceae_unclassified	-1,124780478	0,001211543
g__Parabacteroides	4,068737326	1,74889E-23
g__Paracoccus	1,600603429	1,53622E-06
g__Parvimonas	-1,378879542	0,000161356
g__Pelagibacterium	1,363907087	0,000248877
g__Phocaeicola	-1,012355122	0,012775827
g__Porphyromonas	-1,088686833	0,007775209
g__Prevotella	-1,287679288	0,001655706
g__Rothia	1,685494904	1,58683E-05
g__Scardovia	-1,563074961	8,27518E-06
g__Streptococcus	1,660515289	1,47723E-08
g__Tannerella	-1,662843911	1,10364E-06
g__Treponema	-1,349055889	0,0006751

Table S5. Differentially abundant bacterial genera adjusted for sex, age, smoking, and study centre

Only the genera with q-values less than 0.05, which were significant using ANCOM-BC2's SS filter, are presented in the table.

Genus	Phylum	Mean relative abundance*	Prevalence n (%)*	Log Fold Change	q-value
**Asthma**					
Unclassified *Lachnospiraceae*	*Bacillota*	11.35×10^{-4}	111 (33.13)	-1.11	0.002
GGB1833	*Bacteroidota*	10.73×10^{-4}	64 (19.10)	-1.17	0.002
Unclassified *Candidatus Absconditabacteria*	*Candidatus Absconditabacteriota*	4.56×10^{-4}	87 (25.97)	-1.22	0.002
**Chronic rhinosinusitis**					
Xanthomonas	*Pseudomonadota*	99.05×10^{-4}	49 (14.63)	-1.39	0.047
**FeNO ≥ 25ppb**					
NA	NA	NA	NA	NA	NA

* Mean relative abundance and prevalence is expressed for the entire population (n = 335), even though the number of individuals/samples for the three analyses differ (asthma, n = 333; CRS, n = 334; FeNO, n = 325).

Log Fold Change reflects the difference in bias-corrected abundance of a specific genus between groups expressed on a base-2 logarithmic scale.

Figure S1. Differences in beta diversity between groups

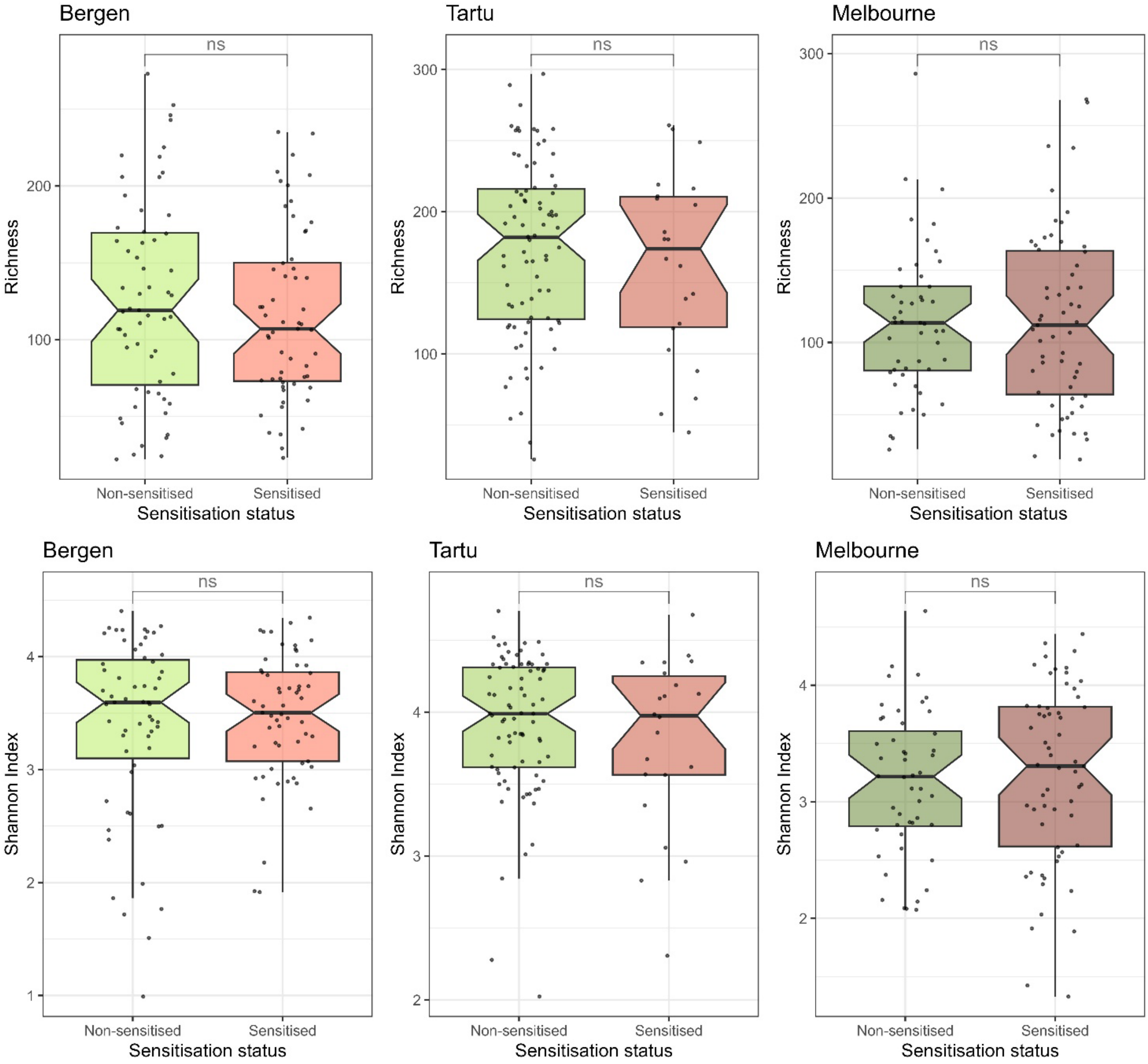

Box plots showing alpha diversity grouped by sensitisation for the three centres. Top row: Richness. Bottom row: Shannon Index. Differences in medians are estimated by Wilcoxon rank-sum test (p-values: ns ≥0.05, * <0.05, ** 0.01, *** <0.001, **** <0.0001).

Figure S2. Differences in beta diversity between groups

(Figure by Clinical Microbiomics)

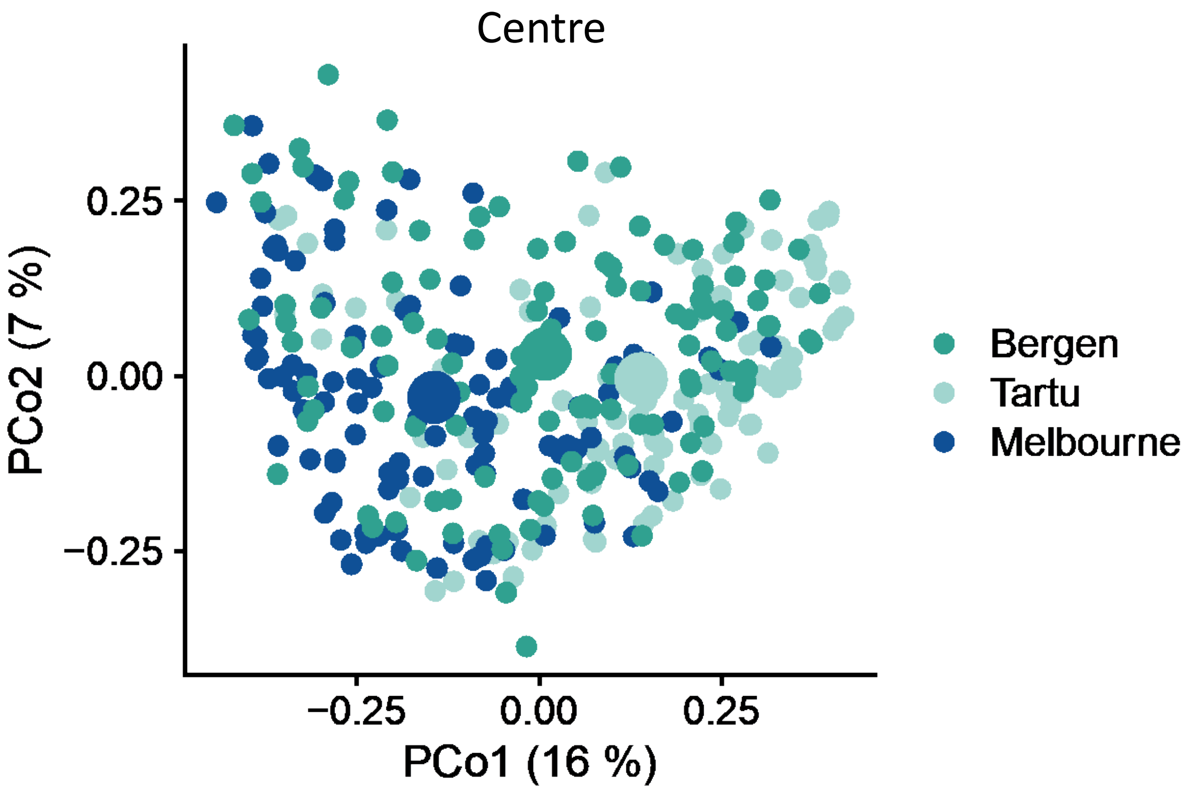

Principal coordinates analysis based on Bray–Curtis dissimilarities among samples, calculated based on the MGS abundances. Samples are color-coded by the centre. The mean (centroid) of samples in each group is indicated with a larger shape. The x- and y-axis labels indicate the microbial variance explained by the first two principal coordinates.

Permutational multivariate analysis of variance (PERMANOVA) tests were performed using the adonis2 function from the vegan R package with 1000 permutations and by = “margin”, thus assessing the marginal effects of the terms (i.e. each marginal term analysed in a model with all other variables).

The role of oral bacteria composition and functional gene profiles in respiratory diseases
Christine Cramer, Ian Philip George Marshall, Michael J. Abramson, Nils Oskar Jõgi, Maryia Khomich, Shyamal Peddada, Bente Sved Skottvoll, Vivi Schlünssen, Randi Jacobsen Bertelsen

Figure S3. Violin plots illustrating the prevalence of the genera and functional genes unique to either cases or non-cases

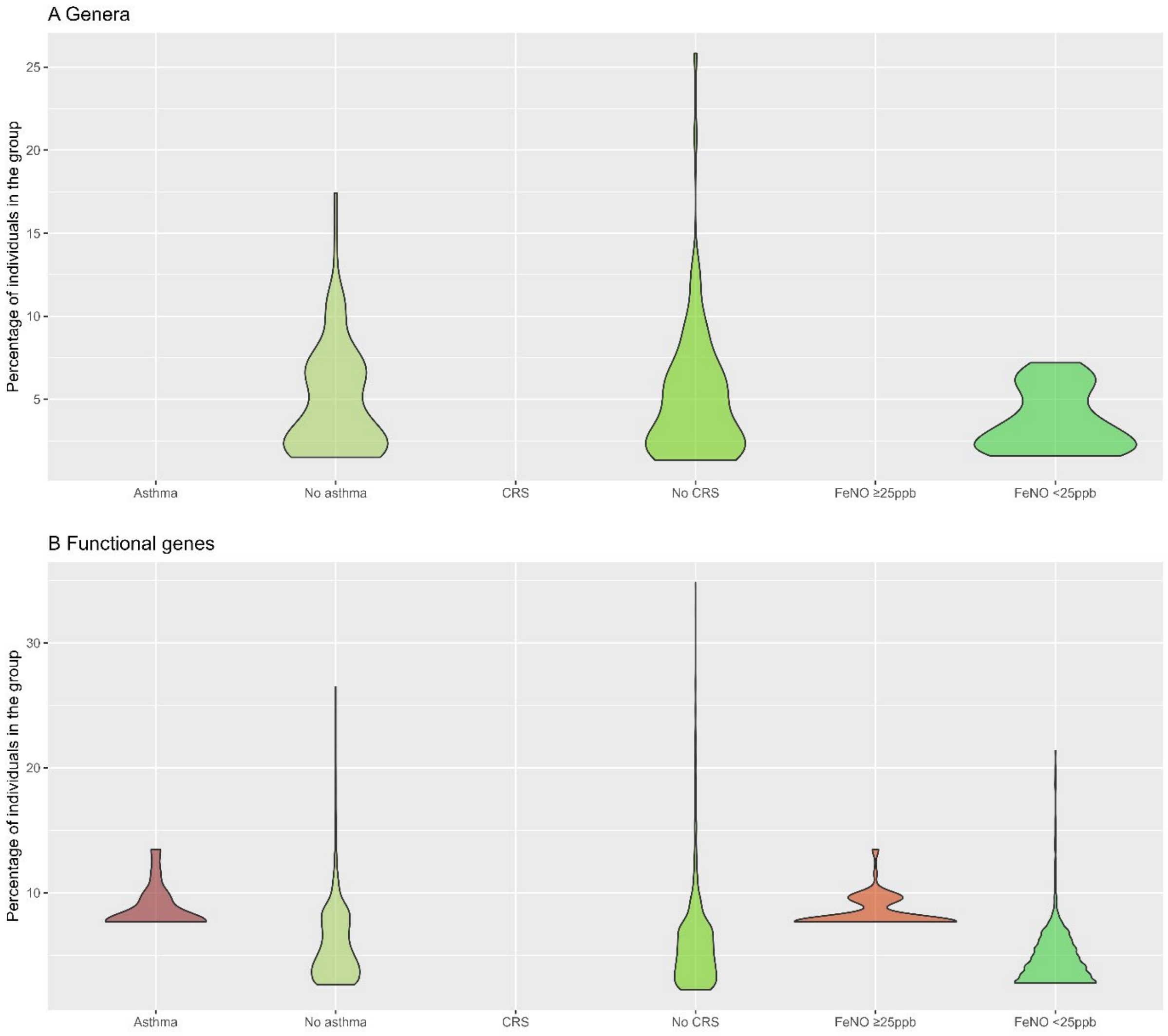

Figure S4. Volcano plots of differentially abundant genera adjusted for sex, age, smoking, and study centre

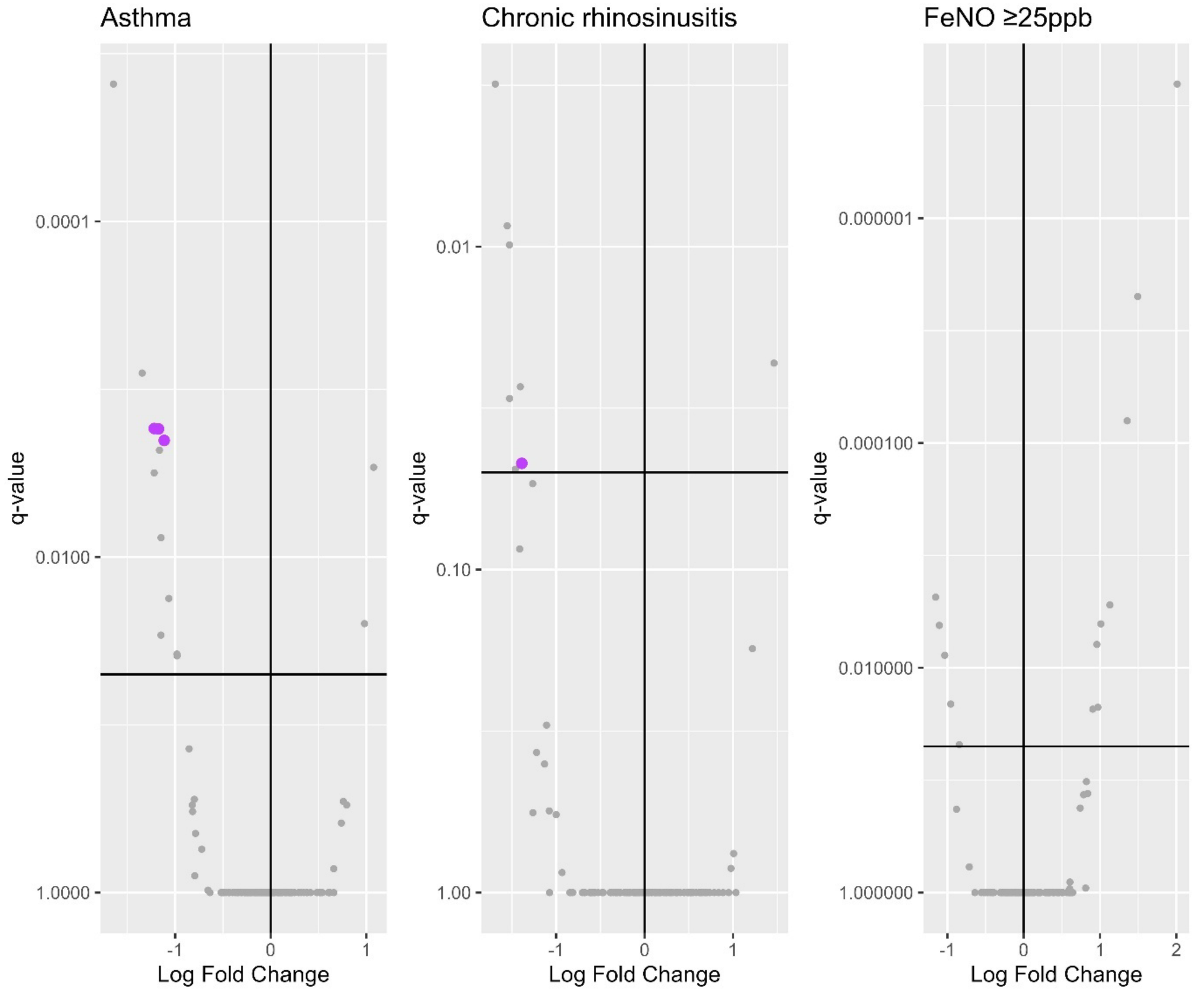

The data points above the x-axis represent genera significantly differentially abundant (q-value less than 0.05). The purple data points represent genera that were significant using ANCOM-BC2's SS filter. Data points to the left of the y-axis represent genera more abundant in non-cases, and data points to the right of the y-axis represent genera more abundant in cases. Log Fold Change reflects the difference in bias-corrected abundance of a specific genera between groups expressed on a base-2 logarithmic scale.

Figure S5. Volcano plots of differentially abundant functional genes adjusted for sex, age, smoking, and study centre

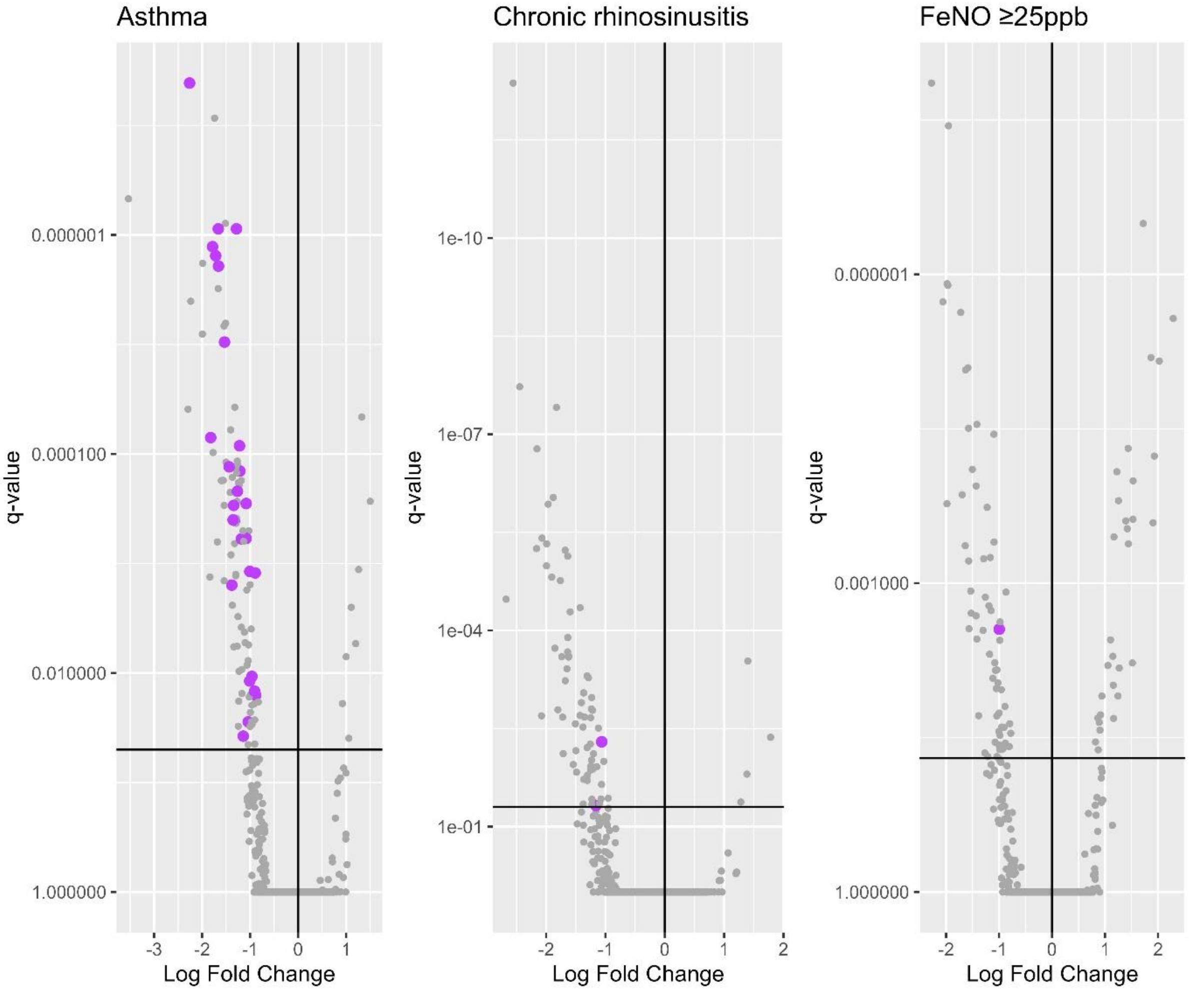

The data points above the x-axis represent functional genes significantly differentially abundant (q-value less than 0.05). The purple data points represent functional genes that were significant using ANCOM-BC2's SS filter. Data points to the left of the y-axis represent functional genes more abundant in non-cases, and data points to the right of the y-axis represent functional genes more abundant in cases. Log Fold Change reflects the difference in bias-corrected abundance of a specific functional genes between groups expressed on a base-2 logarithmic scale.
